# Substrate metabolism regulated by Sestrin2–mTORC1 alleviates pressure overload-induced cardiac hypertrophy in aged heart

**DOI:** 10.1016/j.redox.2020.101637

**Published:** 2020-07-09

**Authors:** Nanhu Quan, Xuan Li, Jingwen Zhang, Ying Han, Weiju Sun, Di Ren, Qian Tong, Ji Li

**Affiliations:** a Department of Cardiovascular Center, The First Hospital of Jilin University, Changchun, 130021, China; b Department of Physiology and Biophysics, University of Mississippi Medical Center, Jackson, MS, 39216, USA; c Department of Surgery, Morsani College of Medicine, University of South Florida, Tampa, FL, 33612, USA

**Keywords:** Sestrin2, Aging, mTORC1, Hypertrophy, Pressure overload

## Abstract

Sestrin2 (Sesn2) is a stress sensor for the mammalian target of rapamycin complex 1 (mTORC1) pathway. Aging impairs cardiac mTORC1 activation, thereby sensitizing the heart to hypertrophy. C57BL/6 J young wild-type (young WT; 4–6 months), aged WT (24–26 months), and young Sestrin2 knockout mice (Y-Sesn2 KO; 4–6 months) underwent transverse aortic constriction (TAC) for pressure overload. Cardiac expression of Sesn2 decreased with age. At 4 weeks after TAC, aged WT and Y-Sesn2 KO exhibited larger hearts and impaired cardiac function, compared with young WT mice. Augmented phosphorylation of mTOR and downstream effectors; damaged mitochondria and elevated redox markers, as well as and impaired glucose and fatty acid oxidation were observed in aged WT and Y-Sesn2 KO hearts. A pressure overload-induced interaction between Sesn2 and GTPase-activating protein activity toward Rags 2 (GATOR2), which positively regulates mTORC1, was impaired in aged WT hearts. Adeno-associated virus 9–Sesn2 treatment rescued Sesn2 expression, attenuated mTORC1 activation, and increased pressure overload tolerance in aged WT and Y-Sesn2 KO hearts. These results indicated that cardiac Sesn2 acts as a pressure overload sensor for mTORC1. Furthermore, Sesn2 deficiency may cause increased sensitivity to hypertrophy in elderly individuals.

## 1 Introduction

Heart failure is an important public health problem. It was recently estimated that 6.2 million Americans ≥20 years of age exhibit heart failure [1]. Moreover, the proportion of people with heart failure in the United States is expected to increase from 2.42% in 2012 to 2.97% in 2030, and the increasing overall cost of heart failure is a serious societal burden [2]. With increasing age, heart failure incidence and mortality increase [1]. Heart failure is the most common cause of hospitalization for patients aged ≥65 years; the incidence of hospitalization for heart failure has increased dramatically from 1% at 40 years of age to 10% at ≥75 years of age [3]. Diuretics and cardiotonic drugs can be used in early stages of treatment to improve symptoms of cardiac insufficiency; ventricular remodeling can be prevented and improved by angiotensin-converting enzyme inhibitors or beta-blockers [4]. End-stage cardiac resynchronization therapy and heart transplantation are suitable treatments, but are expensive and limited to patients with end-stage heart failure [5]. Multiple factors can increase the risk of heart failure, such as the presence of hypertension, diabetes, metabolic syndrome, or atherosclerosis. Among these factors, hypertension is the main pathological factor that contributes to the development of heart failure [6,7]. Heart failure caused by hypertension is characterized by cardiac hypertrophy. Aged myocardium exhibits significantly reduced tolerance to stress; notably, aged heart tissue is reportedly more prone to myocardial hypertrophy when exposed to stress [8]. Therefore, it is important to explore the mechanisms underlying age-related myocardial susceptibility to stress and identify appropriate prevention and treatment strategies.

Myocardial metabolic defects are presumed to be the main sources of age- and stress-related myocardial hypertrophy [9]. Glucose/fatty acid oxidation imbalance could accelerate heart failure in a pressure overload model [10]. The mammalian target of rapamycin (mTOR) is an evolutionally conserved serine/threonine protein kinase, which serves as a key regulator of myocardial metabolism [11]; moreover, it is important in the regulation of cell growth and responding to changes in energy status [[12], [13], [14]]. mTOR can be activated by pathological stimulation to cause cardiac hypertrophy, although it can be partially suppressed by rapamycin [12,13,15]. Furthermore, mTOR inhibitors have been used as immunomodulators for patients who have undergone organ transplantation, as well as patients who have undergone cancer treatment. Rapalogs have been clinically approved for use as antiproliferative treatment; moreover, they reduce the restenosis rate of stents after implantation, and are widely used in treatment of patients with coronary artery disease [11,16]. In addition, microarray analysis indicated the suppression of mTOR pathway in myocardium was protective against atrial remodeling [17]. Finally, sirolimus (i.e., rapamycin) can inhibit cardiac hypertrophy in patients who are undergoing renal transplantation [18]. However, because of its considerable side effects, rapamycin treatment cannot be used regularly for patients with heart failure [16].

mTOR is functionally composed of two catalytic subunits, mTOR-containing multiprotein complex-1 (mTORC1) and mTORC2 [14]. mTORC1 mainly regulates protein and lipid synthesis metabolism, cell growth, mitochondrial biosynthesis, and autophagy; in contrast, mTORC2 mainly regulates cell survival and cytoskeleton [[19], [20], [21]]. Notably, mTORC1 contributes to regulation of hypertrophic heart disease through interactions with eukaryotic initiation factor 4E (eIF4E) binding protein 1 (4EBP1) and eIF4E, which influence initiation of translation. mTORC1 also enhances translation initiation, extension, and protein synthesis by activating S6 kinase (S6K) and S6 [22]. These important mechanisms of myocardial hypertrophy are necessary to maintain cardiac function in the early stages of pressure overload-induced hypertrophy [23]. However, improper control of mTORC1 activity during these early stages can lead to hypertrophic cardiomyopathy. Inhibition of mTORC1 or reduction of mTORC1 substrate activity can facilitate prevention of cardiac hypertrophy [12,13,19,24]; recently, inhibition of mTORC2 activity has also been shown to prevent hypertension and kidney injury in a rat model [25]. Therefore, inhibition of mTORC1 is an important approach for prevention of cardiac hypertrophy.

Sestrins are highly conserved stress response proteins; the three sestrins (Sesn1–3) expressed in mammals are encoded by three independent genomic loci. Sestrins are mainly induced by hypoxia, oxidative stress, DNA damage, and other stress [[26], [27], [28], [29]]; they have also been shown to mediate exercise benefits and adaptations in both fly and mouse models [30]. Recent studies have shown that Sesn2 can inhibit mTORC1 through 5′-adenosine monophosphate-activated protein kinase (AMPK)-dependent or -independent pathways [27,28,[31], [32], [33], [34]]. Sesn2 promotes the tuberous sclerosis complex 2 (TSC2) phosphorylation and Raptor through an AMPK-dependent pathway, thereby inhibiting mTORC1. The AMPK-independent Sesn2–GTPase-activating protein activity toward Rags (GATOR)–mTORC1 pathway can also inhibit mTORC1 [[33], [34], [35]]. Our previous study revealed that Sesn2 expression decreased with age, and that the reduction of Sesn2 protein expression in aged myocardium could cause damage to the Sesn2–AMPK–peroxisome proliferator-activated receptor-gamma coactivator 1-alpha (PGC1α) pathway during ischemia-reperfusion; these changes eventually led to increased myocardial intolerance to ischemia and more serious cardiac insufficiency in aged myocardium [36,37]. We previously found that Sesn2 expression in the heart gradually decreased with age, which suggests that Sesn2 is an age-related protein, recently showed that sestrins protect against aging-related sarcopenia in mice [38,39]. However, it remains unknown whether the reduction of Sesn2 expression in aged heart tissue is related to the incidence and mortality of heart failure. The role of Sesn2 in cardiac hypertrophy is also unclear. This study was performed to investigate whether sestrin2 modulation of cardiac mTORC1 activation is impaired in aging, thereby inducing cardiac hypertrophy.

In the present study, we show that Sesn2 plays an important role in the regulation of cardiac hypertrophy. Furthermore, we show that adeno-associated virus 9 (AAV9)-Sesn2 transfection can partially reverse cardiac hypertrophy in aged wild-type (WT) mice. These findings provide new insight into the mechanism of heart failure and may facilitate new treatments, such as supplementation of exogenous Sesn2 or adeno-associated viral delivery of a constitutively active form of the gene encoding Sesn2, to reduce the incidence of stress-related heart failure in elderly individuals.

## 2 Materials and methods

### 2.1 Animals

Young (4–6 months old; female or male) and aged (24–26 months old; female or male) C57BL/6 J mice were purchased from Charles River (Wilmington, MA, USA), and young Sesn2 KO mice (Y-Sesn2 KO; C57BL/6 J background, 4–6 months old; female or male) were generated as previously described [27,31,40]. University of Mississippi Medical Center and University of South Florida Institutional Animal Care and Use Committees approved the animal study protocol. All animal experiments were performed in compliance with NIH guideline.

### 2.2 TAC procedure and rapamycin treatment

The mouse model of pressure overload was established by TAC surgery under anesthesia with 2% isoflurane, as described previously [12]. Briefly, mice were intubated and their lungs were ventilated under a dissecting microscope, using a small-animal respirator (Harvard Apparatus, Holliston, MA, USA) set at a rate of 135 breaths/min and a tidal volume of 120 ml/100 g body weight. A blunted 7-0 suture was wrapped below the aortic arch, between the brachiocephalic trunk and left common carotid artery. A 7-0 suture was used to tie a shortened and blunted 27-G needle onto the aortic arch. After the needle had been tightly secured, it was removed from the knot, leaving the aorta permanently constricted. The chest cavity was sutured closed in layers, using a 5–0 absorbable suture. The mice were slowly weaned off the ventilator, kept warm, and returned to their cages. Medications for pain relief were administered postoperatively. Rapamycin (Sigma-Aldrich, St. Louis, MO, USA; 2 mg kg^−1^ d^−1^) or vehicle was administered intraperitoneally to TAC or sham-operated mice, as previously described [12]. The solvent for rapamycin was 0.5% dimethylsulfoxide; vehicle was 0.5% dimethylsulfoxide.

### 2.3 Adeno-associated viral delivery

Viral delivery was performed in accordance with previously published methods [38]. Briefly, mice were anesthetized with isoflurane (2%) and placed on a ventilator. For each mouse, the chest was entered from the left side through the fourth intercostal space. After dissection of the aorta and pulmonary artery, AAV9 (pAAV-G-GMV)-Sesn2 (5 × 10^7^ genome copies per mouse; Cat. No. AAVP0205933; Applied Biological Materials Inc., Richmond, Canada) was injected into the left ventricular cavity through a 27-G catheter over 50 s, while the aorta and pulmonary artery were transiently crossed-clamped. In sham-operated animals, normal saline (50 μl) was injected into the left ventricular cavity over 50 s, while the aorta and pulmonary artery were crossed-clamped. This procedure allowed the solution containing AAV9 to circulate through the coronary arteries and perfuse the heart without direct manipulation of these arteries. After 50 s, clamps on the aorta and pulmonary artery were released. After removal of air and blood, the chest was closed; each mouse was extubated and transferred back to its cage.

### 2.4 Histological analysis

Histological analysis was performed in accordance with previously published methods [41]. For histological analysis, hearts were arrested with a 10% potassium chloride solution at end-diastole, then fixed in 4% paraformaldehyde. Fixed hearts were embedded in paraffin and cut transversely into 5-μm sections. Serial heart sections were stained with hematoxylin-eosin or wheat germ agglutinin (#W11261; Invitrogen, Carlsbad, CA, USA) to measure myocyte cross-sectional areas. Sections were stained with Masson's trichrome (collagen, blue; cytoplasm, red/pink) for collagen deposition analysis.

### 2.5 Echocardiography

Echocardiography was performed in accordance with previously published methods [36]. Mice were anaesthetized (isoflurane) and transthoracic M-mode echocardiography (Vevo3100; VisualSonics, Toronto, Canada) was performed to evaluate cardiac function. M-mode tracings were recorded from the short axis of the left ventricle at the level of the papillary muscles. The left ventricle end-diastolic dimension and left ventricle end-systolic dimension were measured at the largest and smallest left ventricle areas, respectively. Simpson's measurements were performed to obtain averaged ejection fraction (EF) and fractional shortening (FS).

### 2.6 Immunoblotting

Immunoblotting and immunoprecipitation were performed as previously described [38]. For immunoblotting, protein concentrations were measured using the Bradford method (Bio-Rad, Hercules, CA, USA). Cardiomyocyte lysate proteins were separated by sodium dodecyl sulfate-polyacrylamide gel electrophoresis and transferred to polyvinylidene difluoride membranes (Millipore, Billerica, MA, USA). Membranes were blocked for 1 h at room temperature (23–25 °C) in 5% milk in Tris-buffered saline with Tween-20. They were then incubated with primary antibody at 4 °C overnight; primary antibodies were diluted in 5% milk in Tris-buffered saline with Tween-20, and antibody details are listed in Expanded View Table 4. Membranes were washed three times for 5 min each in Tris-buffered saline with Tween-20 at room temperature (23–25 °C), then incubated with secondary antibody (horseradish peroxidase-conjugated goat anti-rabbit IgG, 1:5000, Boster Bio (Wuhan, China) for 1 h at room temperature (23–25 °C). Finally, they were developed using the enhanced chemiluminescence method. For immunoprecipitation, isolated myocardial tissue (whole heart) was homogenized and lysed in standard lysis buffer with phenylmethylsulfonyl fluoride and a protease inhibitor cocktail. Anti-Sesn2 antibody (2 μg) and immunoprecipitation beads (10–50 μl; Protein G Sepharose beads 4 Fast Flow, Cat. No. 17-0618-02; GE Health Care, Waukesha, WI, USA) were added to the lysate; the lysate was then incubated with gentle shaking at 4 °C overnight. Subsequently, immunoprecipitates were collected, washed with standard lysis buffer, and then subjected to immunoblotting as above.

### 2.7 Glucose uptake and glycolysis analysis

Glucose uptake and glycolysis were processed as previously described [41]. Glucose uptake and glycolysis were analyzed in the Langendorff heart perfusion system by measuring the production of ^3^H_2_O from D-[2–^3^H]glucose or D-[5-^3^H]glucose, respectively. Ten minutes before they were anesthetized, mice received intraperitoneal heparin (100 units). Isolated hearts were then retroperfused by using the Langendorff perfusion system (Radnoti, Monrovia, CA, USA) with Krebs–Henseleit buffer that contained 7 mM glucose, 1% bovine serum albumin, 0.4 mM sodium oleate, 10 mU/ml insulin, and D-[2–^3^H]glucose/D-[5-^3^H]glucose, bubbled with 95% O_2_/5% CO_2_. The system was maintained at 37 °C. For both glucose uptake and glycolysis measurements, isolated hearts were subjected to 20 min of basal perfusion, followed by 10 min global no-flow ischemia, then 20 min of reperfusion. Perfusate was recycled and collected at 5-min intervals to measure its radioactivity. Metabolized ^3^H_2_O was separated from D-[2–^3^H]glucose or D-[5-^3^H]glucose by filtration through anion-exchange 1-X8 resin (Bio-Rad). Rates of glucose uptake and glycolysis were calculated by the amount of ^3^H_2_O production. Ten milliliters of scintillation fluid were added to each vial, then mixed well. Radioactive signals were measured on a liquid scintillation counter.

### 2.8 Oleate/glucose oxidation analysis

Oleate/glucose oxidation analysis was performed as previously described [38]. The working heart preload was set at 15 cmH_2_O, and the afterload was set at 80 cmH_2_O.The flow rate was maintained at 15 ml/min. Heart function was monitored by a pressure transducer connected to the aortic outflow [9,10].-^3^H-oleate (50 mci/L) and ^14^C-glucose (20 mci/L)–labeled bovine serum albumin buffer was perfused into the heart via the pulmonary vein, then pumped out through the aorta. Perfusate that had been pumped out from the aorta and outflowed from coronary venous artery was recycled and collected at 5-min intervals to test its radioactivity. The fatty acid level was determined by the production of ^3^H_2_O from Ref. [9,10]-^3^H-oleate. Metabolized ^3^H_2_O was separated from Ref. [9,10]-^3^H-oleate by filtration through an anion-exchange resin (Bio-Rad). Glucose oxidation was measured and sampled at 5-min intervals by metabolized ^14^CO_2_ that was dissolved in the perfusate buffer and by gaseous ^14^CO_2_, which was further dissolved in sodium hydroxide. Sulfuric acid was added to perfusate samples to release ^14^CO_2_, to separate it from ^14^C-glucose. ^3^H and ^14^C signals were measured to discriminate metabolic products from fatty acid and glucose, respectively.

### 2.9 mRNA analysis by quantitative polymerase chain reaction (qPCR)

Mouse heart tissues (left ventricle) were collected for RNA extraction using TRIzol® reagent (Invitrogen). mRNA was reverse transcribed into double-stranded cDNA fragments using the Thermo Script RT-PCR system (Invitrogen), with 1.5 μg total RNA and 1 μl reverse transcriptase, in accordance with the manufacturer's instructions. qPCR was performed using 20-μl reactions, which contained 12.5 ng cDNA, 330 nM each for forward and reverse primers, and 10.5 ng SYBR Green Supermix (Bio-Rad). A thermocycler (CFX96 Touch PCR; Bio-Rad) was used for amplification with the following protocol: 95 °C for 10 min, followed by 35 cycles of 95 °C for 10 s and 60 °C for 45 s. For each target gene, a standard curve was generated and the starting quantity of mRNA was calculated using Bio-Rad qPCR detection system software. All transcripts were analyzed in duplicate and normalized to β-actin. The delta delta Ct method was used to analyze the results. Primer sequences for qPCR are provided in Expanded View Table 4.

### 2.10 Relative quantification of mitochondrial DNA copy number

Mouse heart tissues (whole heart) were collected with RNase A (Invitrogen). The mitochondrial DNA content relative to nuclear DNA was assessed by qPCR, using the protocol described above for mRNA analysis.

### 2.11 Transmission electron microscopy

Transmission electron microscopy was performed as previously described [38]. Briefly, heart tissues were rapidly immersed in tissue fixative buffer (10% buffered formaldehyde, pH 7.4, Carson-Millonig formulation; RI31911; Ricca Chemicals, Arlington, TX, USA) at 4 °C for 8 h. After fixation, samples were cut into 70-nm sections using an ultramicrotome, then placed on transmission electron microscopy grids, stained with lead citrate, and imaged using a Tecnai G2 spirit Twin transmission electron microscope (FEI Company, Hillsboro, OR, USA) at 80 kV.

### 2.12 Statistical analysis

Data are reported as the mean ± standard error of the mean. The numbers of experiments in each group are presented in the corresponding figures and figure legends. Two-tailed Student's *t*-test, two-way ANOVA using Tukey's test for *post hoc* comparisons GraphPad Prism 8.0 (GraphPad Software, La Jolla, CA, USA). Differences with p < 0.05 were considered statistically significant.

## 3 Results

### 3.1 Aging-related Sesn2 deficiency aggravates cardiac hypertrophy in a manner observed during aging

**Fig. 1:**
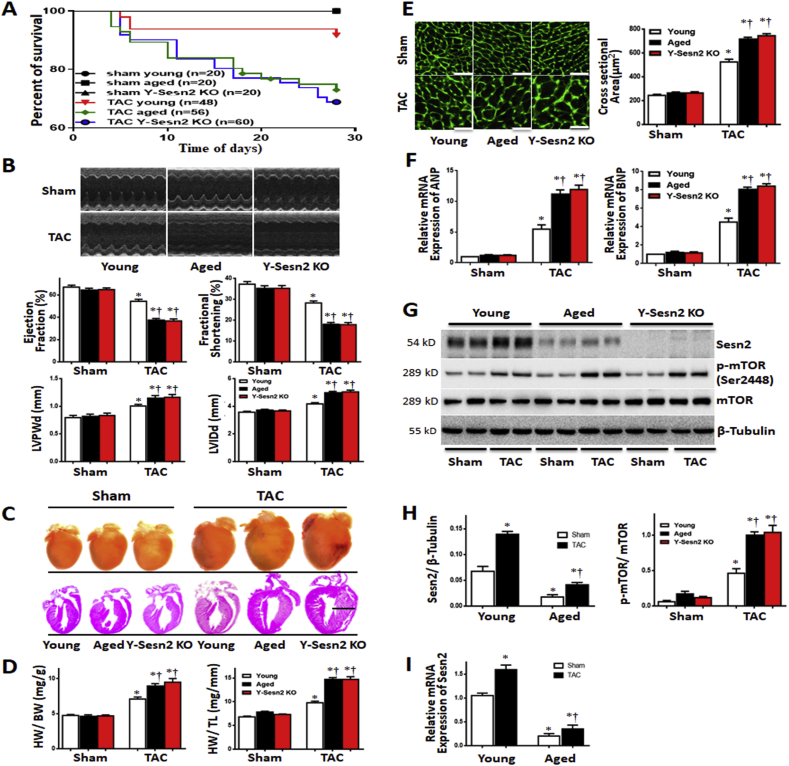
Aged WT and Y-Sesn2 KO hearts show similar responses to pressure overload induced by transverse aortic constriction (TAC). (A) Percent survival rates for young WT(Young), aged WT(Aged), and Y-Sesn2 KO mice subjected to sham operation or TAC. Sample size per group noted in figure. (B) Representative echocardiography results for Y-Sesn2 KO, aged WT, and young WT mice at 4 weeks after TAC or sham surgery. The EF, FS, left ventricular posterior wall at end-diastole (LVPWd), and left ventricular internal diameter at end-diastole (LVIDd) were quantified from echocardiography (n = 15–20 per group). (C) Representative images of whole hearts (scale bar, 2 mm) and hematoxylin–eosin (scale bar, 2 mm) staining images of Y-Sesn2 KO, aged WT, and young WT mice at 4 weeks after TAC or sham surgery. (D) Heart weight/body weight (HW/BW) ratios and heart weight/tibial length (HW/TL) ratios of Y-Sesn2 KO, aged WT, and young WT mice at 4 weeks after TAC or sham surgery (n = 15–20 per group). (E) Representative wheat germ agglutinin (scale bars, 50 μm) staining images and quantification of cardiomyocyte size in Y-Sesn2 KO, aged WT, and young WT mice at 4 weeks after TAC or sham surgery (n = 30–40 per group). (F) mRNA expression analysis of ANP and BNP in Y-Sesn2 KO, aged WT, and young WT mice at 4 weeks after TAC or sham surgery (n = 5–6 per group). (G) Immunoblot for the indicated proteins from hearts of Y-Sesn2 KO, aged WT, and young WT mice at 4 weeks after TAC or sham surgery. (H) Quantification of Sesn2 protein in aged WT and young WT mice at 4 weeks after TAC or Sham surgery, as well as quantification of phospho-mammalian target of rapamycin (p-mTOR)/mTOR expression in Y-Sesn2 KO, aged WT, and young WT mice at 4 weeks after TAC or sham surgery (n = 5–6 per group). (I) Relative Sesn2 mRNA expression levels in aged WT and young WT mice at 4 weeks after TAC or sham surgery. (n = 5–6 per group). Values are mean ± SEM, *p < 0.05 vs. Sham group; ^†^p < 0.05 vs. corresponding young WT group.

To investigate the relationship between Sesn2 and aging during cardiac hypertrophy, young WT (Young), aged WT (Aged) and Y-Sesn2 KO mice were subjected to transverse aortic constriction (TAC) surgery and their functional cardiac phenotypes were evaluated. Under basal conditions, there were no differences in mortality (Fig. 1A), cardiac contractile function (Fig. 1B), heart size (Fig. 1C), heart weight/body-weight, heart weight/tibia length (Fig. 1D), cardiomyocyte size (Fig. 1E), or expression levels of atrial natriuretic peptide (ANP) and B-type natriuretic peptide (BNP) in heart tissue (Fig. 1F) among young WT, aged WT, and Y-Sesn2 KO mice. Notably, 4 weeks after TAC surgery, mortality was markedly higher among aged WT mice and Y-Sesn2 KO mice than among young WT mice (Fig. 1A); however, mortality did not significantly differ between aged WT and Y-Sesn2 KO mice (Fig. 1A). Echocardiography showed that EF and FS in aged WT and Y-Sesn2 KO mice were reduced after TAC surgery, while left ventricular posterior wall at end-diastole and left ventricular internal diameter at end-diastole were elevated, which indicated impaired cardiac functions in aged WT and Y-Sesn2 KO hearts (Fig. 1B). TAC surgery-induced cardiac hypertrophy and heart weight (normalized to both body weight and tibial length) were significantly increased in aged WT and Y-Sesn2 KO mice, compared with young WT mice (Fig. 1C and D). In addition, wheat germ agglutinin staining was performed to analyze cardiac hypertrophy. Remarkable hypertrophy of myocardial tissues after TAC surgery was observed in aged WT mice and Y-Sesn2 KO mice, compared with young WT mice (Fig. 1E). mRNA levels of ANP and BNP were elevated in aged WT and Y-Sesn2 KO mice after TAC surgery (Fig. 1F). These abnormalities were enhanced in the hearts of aged WT and Y-Sesn2 KO mice, compared with young WT mice (Fig. 1F). These findings suggested that both Sesn2 deficiency and aging could lead to intolerance of the heart to TAC surgery.

Notably, the protein and mRNA levels of Sesn2 were significantly reduced in hearts of aged WT mice, compared with young WT mice. Sesn2 expression was absent in Y-Sesn2 KO mice. After TAC surgery, Sesn2 protein and mRNA expression levels significantly increased in hearts of young WT mice, while they increased slightly in aged WT mice (Fig. 1G and H). These results suggested that the reduced Sesn2 observed in aged hearts may contribute to their intolerance to cardiac hypertrophy.

Activation of mTOR serves as the main mechanism of pressure overload-induced cardiac hypertrophy [12,13]; this signaling pathway is also involved in age-related hypertrophy [42]. Therefore, we analyzed core mTOR pathway-related regulators in the hearts of young WT, aged WT, and Y-Sesn2 KO mice. In all three types of mice, pressure overload caused elevation of myocardial p-Akt(Ser^473^), which is generally regarded as an upstream target of mTORC1 (Expanded View Fig. 1A and B). S6 and 4EBP1 are two well-defined downstream targets of mTORC1; activation of mTORC1 increases p-S6 and p-4EBP1 levels. Notably, Sesn2 deficiency and aging caused significant overactivation of mTORC1, S6, and 4EBP1, compared with young WT mice. However, activation of AKT did not change in aged and Y-Sesn2 KO hearts (Expanded View Fig. 1A and B). These results suggest that both Sesn2 deficiency and aging can enhance the pressure overload-induced activation of mTORC1.

### 3.2 Sesn2 ameliorates cardiac hypertrophy by inhibiting mTOR

**Fig. 2:**
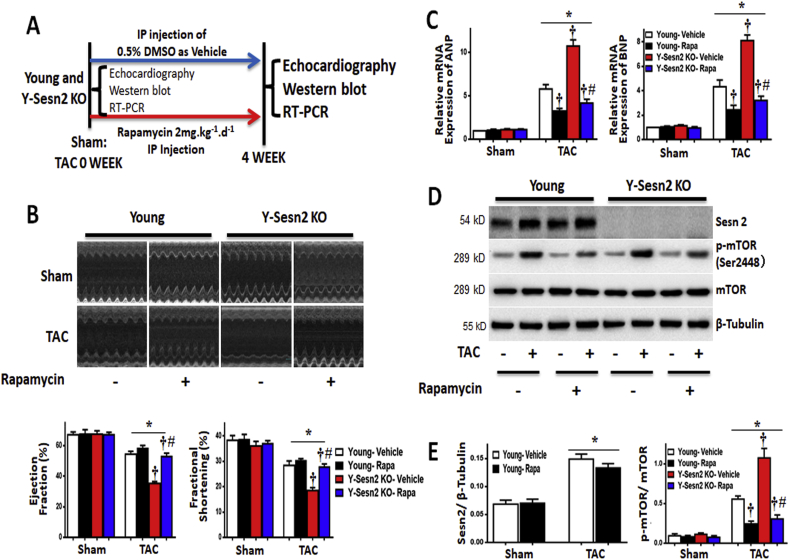
Inhibition of mTOR reduced cardiac hypertrophy induced by pressure overload. (A) Experimental design: Y-Sesn2 KO mice and young WT (Young) littermates were treated with intraperitoneal (IP) rapamycin or dimethylsulfoxide (DMSO, vehicle). Hearts of Y-Sesn2 KO and young WT mice were monitored by ultrasonography, and harvested 4 weeks after transverse aortic constriction (TAC) for immunoblotting and qPCR. (B) Echocardiography showed that rapamycin treatment improved the resistance of Y-Sesn2 KO hearts to TAC, as demonstrated by EF and FS (n = 5–10 per group). (C) Relative expression analysis of ANP and BNP (n = 5–6 per group). (D) Immunoblot for indicated proteins from hearts of sham or TAC operated young WT or Y-Sesn2 KO mice, treated with or without rapamycin. (E) Quantification of Sesn2 protein in sham or TAC operated young WT mice, treated with or without rapamycin, as well as quantification of phospho-mammalian target of rapamycin (p-mTOR)/mTOR expression in sham or TAC operated young WT or Y-Sesn2 KO mice, treated with or without rapamycin (n = 5–6 per group). Values are mean ± SEM, *p < 0.05 vs. Sham group; ^†^p < 0.05 vs. corresponding young WT group; ^#^p < 0.05 vs. corresponding Y-Sesn2 KO group.

Inhibition of mTOR by rapamycin can lead to cardiovascular improvement, as well as prevention of age-related hypertrophy [42]. However, it has been unclear whether Y-Sesn2 KO mice enhances cardiac hypertrophy through activation of mTOR. To investigate whether inhibition of mTOR caused reduced cardiac hypertrophy in Y-Sesn2 KO mice, we treated young WT mice and Y-Sesn2 KO mice with rapamycin or vehicle at the time of TAC or sham surgery (Fig. 2A).

In young WT (Young) and Y-Sesn2 KO mice that underwent sham surgery, rapamycin treatment had no significant effects on cardiac contractile function (Fig. 2B) or on the expression levels of ANP and BNP (Fig. 2C) in heart tissue. Importantly, TAC -induced reductions of EF and FS in hearts of Y-Sesn2 KO mice were significantly reversed by rapamycin treatment (Fig. 2B). Rapamycin treatment also led to reductions of ANP and BNP mRNA levels in Y-Sesn2 KO and young WT hearts after TAC surgery (Fig. 2C). With rapamycin treatment, young WT and Y-Sesn2 KO hearts had similar mRNA levels of ANP and BNP after TAC (Fig. 2C). Next, we investigated activation of the mTOR pathway. Rapamycin had no significant effect on Sesn2 protein expression in young WT hearts. Similar to the findings regarding ANP and BNP expression, rapamycin treatment reduced the activities of mTOR, S6, and 4EBP1 in young WT and Y-Sesn2 KO hearts, following TAC surgery. With rapamycin treatment, young WT and Y-Sesn2 KO hearts exhibited similar activities of mTOR, 4EBP1, and S6 after TAC (Fig. 2D and E; Expanded View Fig. 2A and B). However, AKT activation was not affected by rapamycin, which is potentially because AKT is upstream of mTOR. These results suggested that Y-Sesn2 KO enhanced cardiac hypertrophy due to overactivation of the mTORC1 pathway.

**Fig. 3:**
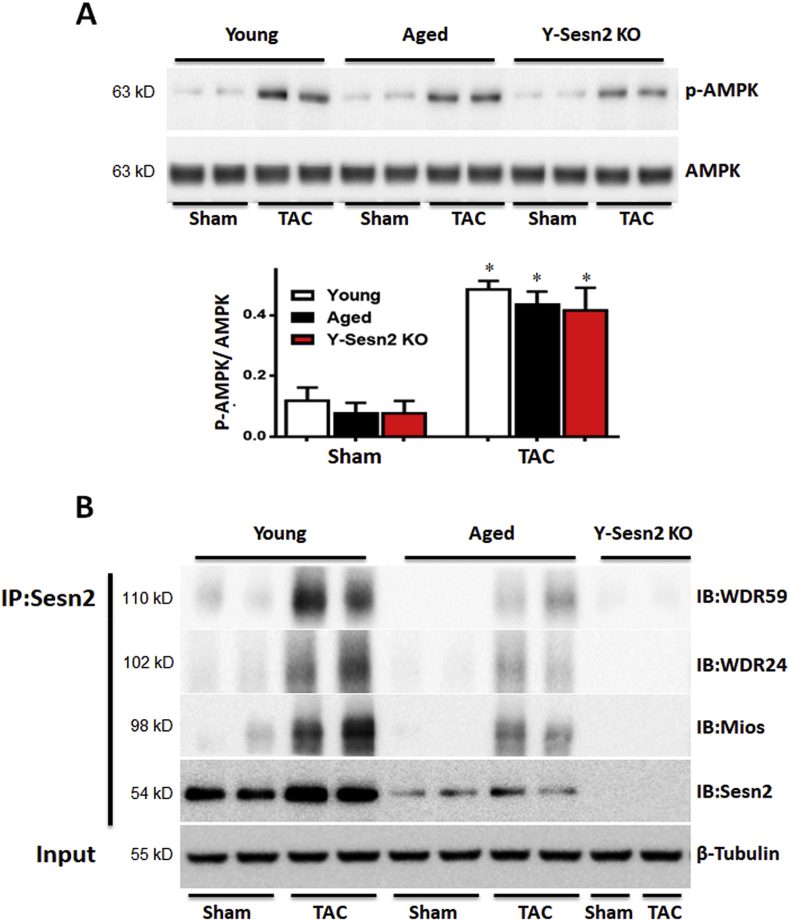
Sesn2 interacts with MIOS, WDR24, and WDR59 of the GATOR2 complex to inhibit mTORC1. (A) Immunoblot for p-AMPK and AMPK proteins from hearts of Y-Sesn2 KO, aged WT(Aged), and young WT (Young) mice at 4 weeks after TAC or sham surgery (n = 6 per group). (B) Heart tissues were harvested from young WT and aged WT mice that had been subjected to TAC or sham surgery. Nuclear extracts were isolated and immunoprecipitated (IP) with anti-Sesn2 antibody, then immunoblotted (IB) with anti-Sesn2, anti-WDR59, anti-WDR24, or anti-MIOS antibodies. Values are mean ± SEM, *p < 0.05 vs. Sham group.

Previous studies showed that Sesn2 could inhibit mTORC1 through both AMPK-dependent and -independent pathways [27,34]. To explore the mechanisms by which Sesn2 inhibits mTOR, we assayed the activities of AMPK in the hearts of young WT, aged WT, and Y-Sesn2 KO mice that had undergone TAC or sham surgery. Immunoblotting analysis showed that AMPK activation was not affected by the absence of Sesn2 or by aging (Fig. 3A). mTOR may also be inhibited via Sesn2 protein binding to members of the GATOR2 protein complex (i.e., MIOS, WDR24, WDR59) in the AMPK-independent pathway. GATOR2 can regulate the activity of RagB, a small GTPase essential for mTORC1 activation [35]. To verify whether Sesn2 inhibits mTORC1 through binding interactions with MIOS, WDR24, and/or WDR59 in the GATOR2 complex, a Co-immunoprecipitation assay was performed. The results demonstrated that MIOS, WDR24, and WDR59 were all present in Sesn2-immunoprecipitated complexes from both young and aged hearts of mice that had undergone TAC surgery (Fig. 3B). In summary, Sesn2 interacted with MIOS, WDR24, and WDR59 of the GATOR2 complex to inhibit mTORC1.

### 3.3 Sesn2 contributes to substrate metabolism in response to pressure overload

Sesn2 knockout or aging enhances pressure overload-induced cardiac hypertrophy through excessive activation of mTOR, which is the key regulator of myocardial metabolism. During the development of cardiac hypertrophy, excessive activation of mTOR leads to increased mitochondrial damage, as well as reductions in glucose metabolism, fatty acid metabolism, and oxygen metabolism [43]. Accordingly, we evaluated glucose metabolism, fatty acid metabolism, and oxygen metabolism in hearts of mice that had undergone TAC or sham surgery.

**Fig. 4:**
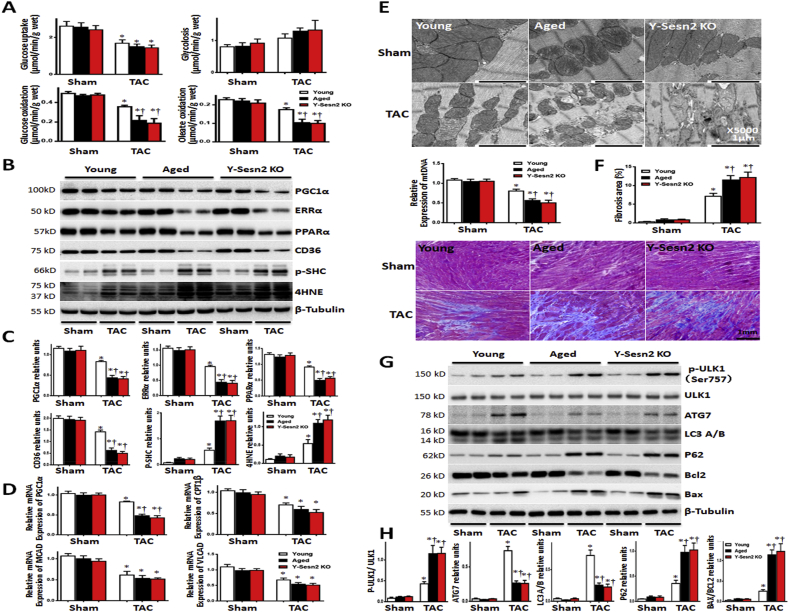
Regulation of substrate metabolism and autophagy in response to pressure overload. (A) Glucose uptake, glycolysis, glucose oxidation, and oleate oxidation in hearts from Y-Sesn2 KO, aged WT (Aged), and young WT (Young) mice at 4 weeks after TAC or sham surgery (n = 5–6 per group). (B) Immunoblot for indicated proteins from hearts of Y-Sesn2 KO, aged WT, and young WT mice at 4 weeks after TAC or sham surgery. (C) Quantification of relative expression levels of indicated proteins (n = 5–6 per group). (D) Relative mRNA expression analysis of PGC1α, carnitine palmitoyltransferase (CPT)1β, medium-chain acyl-CoA dehydrogenase (MCAD), and very-long-chain acyl-CoA dehydrogenase (VLCAD) in Y-Sesn2 KO, aged WT, and young WT mice at 4 weeks after TAC or sham surgery (n = 5–6 per group). (E) Representative transmission electron microscopy images of mitochondrial morphology in Y-Sesn2 KO, aged WT, and young WT hearts, as well as mitochondrial (mt) DNA levels from Y-Sesn2 KO, aged WT, and young WT mice at 4 weeks after TAC or sham surgery (n = 5–6 per group). (F) Masson Trichrome staining and expression analysis of Y-Sesn2 KO, aged WT, and young WT mice at 4 weeks after TAC or sham surgery (n = 5–6 per group). (G) Immunoblot for indicated proteins from hearts of Y-Sesn2 KO, aged WT, and young WT mice at 4 weeks after TAC or sham surgery. (H) Quantification of relative expression levels of indicated proteins (n = 5–6 per group). Values are mean ± SEM, *p < 0.05 vs. Sham group; ^†^p < 0.05 vs. corresponding WT group.

Glucose uptake, glucose oxidation, and oleate oxidation were suppressed by TAC surgery, whereas glycolysis was not. In addition, glucose uptake and oleate oxidation exhibited greater suppression in hearts of Y-Sesn2 KO mice and aged WT (Aged) mice, compared with young WT (Young) mice; there were no differences between Y-Sesn2 KO mice and aged WT mice (Fig. 4A). Immunoblotting results suggested that the expression levels of mitochondrial synthesis-associated proteins (PGC1α, ERRα, PPARα, and CD36) were reduced in young WT, aged WT, and Y-Sesn2 KO mice after TAC surgery; these reductions were greatest in aged WT and Y-Sesn2 KO mice (Fig. 4B and C). The expression of the glycolysis-related protein, PDH, was not suppressed by TAC. The level of glucose transporter 4 in cell membrane was significantly lower after TAC surgery, which led to the reduction of glucose uptake (Expanded View Fig. 3A and B). These results were consistent with our findings shown in Fig. 4A. Next, qPCR results showed that relative expression of the metabolic gene, PGC1α, was reduced in young WT, aged WT, and Y-Sesn2 KO mice after TAC surgery; this reduction was greater in aged WT and Y-Sesn2 KO mice. The relative expression levels of oleate oxidation-related genes (CPT1β, MCAD, and VLCAD) were also reduced after TAC surgery (Fig. 4D). Oxidative stress can accelerate mitochondrial damage [44]. TAC surgery led to increased expression levels of oxidative stress-related proteins, SHC and 4HNE; these expression levels were higher in Y-Sesn2 KO and aged hearts than in young hearts (Fig. 4B and C). To examine the effects of Y-Sesn2 KO and aging on mitochondrial structure and physiologic function, transmission electron microscopy was used to observe mitochondrial morphology in young WT, aged WT, and Y-Sesn2 KO hearts. Significant mitochondrial fission was observed in aged and Y-Sesn2 KO hearts, compared with young hearts (Fig. 4E). In addition, the levels of fibrosis were significantly greater in aged WT and Y-Sesn2 KO hearts than in young hearts (Fig. 4F). Inadequate repair after pressure overload-induced cardiac hypertrophy may be caused by metabolic disorder. The above results suggest that Sesn2 knockout and aging induced disordered mitochondrial synthesis and oxidative stress-related damage, which may hinder glucose oxidation and oleate oxidation metabolism.

In addition, mTOR serves to inhibit autophagy [14]. However, it is unclear whether Sesn2 knockout and aging could inhibit autophagy by promoting mTOR. To explore the effects of Sesn2 knockout and aging on autophagy during pressure overload-induced cardiac hypertrophy, the expression levels of a series of autophagy-related proteins were detected by immunoblotting. Significantly elevated phosphorylation of autophagy-related kinase UNC-51–like kinase 1(ULK1) at Ser^757^ was observed in aged WT and Y-Sesn2 KO hearts in response to pressure overload. The expression levels of autophagy-related proteins (ATG7 and LC3A/B) were significantly reduced in aged WT and Y-Sesn2 KO hearts during cardiac hypertrophy, while levels of apoptosis-related proteins (BCL2 and BAX) increased (Fig. 4G and H). These findings indicated that Sesn2 knockout and aging both inhibit autophagy during pressure overload-induced cardiac hypertrophy.

### 3.4 Rescue of Sesn2 ameliorates pressure overload-induced cardiac hypertrophy in aged heart

**Fig. 5:**
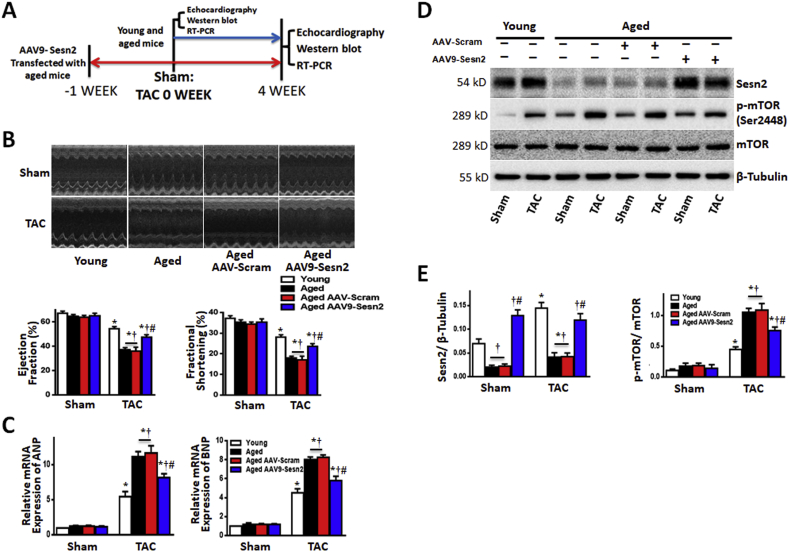
Sesn2 rescue in aged hearts ameliorates pressure overload-induced cardiac hypertrophy. (A) Experimental design: aged WT(Aged) mice were treated with AAV9-Sesn2 at 1 week before TAC. Heart function was monitored by ultrasonography and heart tissues were harvested at indicated time points for immunoblotting and qPCR. (B) Echocardiography showed that AAV9-Sesn2 treatment improved resistance of aged heart to TAC, as demonstrated by EF and FS (n = 6–15 per group). (C) Expression analysis of ANP and BNP (n = 5–6 per group). (D) Immunoblot for Sesn2, phospho-mammalian target of rapamycin (p-mTOR), and mTOR from hearts of sham or TAC operated young WT and aged WT mice, treated with or without AAV9-Sesn2 as indicated in panel A. (E) Quantification of immunoblot shown in panel D (n = 5–6 per group). Values are mean ± SEM, *p < 0.05 vs. Sham group; ^#^p < 0.05 vs. corresponding young group; ^†^p < 0.05 vs. corresponding aged group.

To verify whether the reduced levels of Sesn2 in aged hearts contributed to their intolerance to cardiac hypertrophy, we used adeno-associated virus 9 (AAV9)-Sesn2 to rescue the impaired Sesn2 level in aged WT (Aged) hearts (Fig. 5A). We verified the level of Sesn2 rescue in Y-Sesn2 KO mice before AAV9-Sesn2 was introduced to aged WT mice. AAV9-Sesn2 treatment was performed 1 week before the TAC or sham surgery, as described in Expanded View Fig. 4A. In young WT (Young) and Y-Sesn2 KO mice that underwent sham surgery, AAV9-Sesn2 exhibited minimal effects on cardiac systolic function (Expanded View Fig. 4B) and expression levels of ANP and BNP in heart tissue (Expanded View Fig. 4C). At 2 weeks after TAC, EF and FS both decreased in hearts of Y-Sesn2 KO and Y-Sesn2 KO + AAV9 scram mice, but not in young WT mice or Y-Sesn2 KO + AAV9-Sesn2 mice. At 4 weeks after TAC, EF and FS decreased further; however, AAV9-Sesn2 treatment rescued impaired cardiac systolic function in Y-Sesn2 KO mice (Expanded View Fig. 4B). At 2 weeks after TAC, the levels of ANP and BNP were significantly higher in heart tissue of Sesn2 KO and Y-Sesn2 KO + AAV9-scram mice than in heart tissue of WT mice and Y-Sesn2 KO + AAV9-Sesn2 mice. At 4 weeks after TAC, ANP and BNP increased further in all mice, but the relative differences among mice were identical to those at two weeks (Expanded View Fig. 4C). Immunoblotting showed that AAV9-Sesn2 transfection rescued the levels of Sesn2 in heart tissue until 4 weeks after TAC. Moreover, AAV9-Sesn2 could inhibit the excessive activation of mTORC1, S6, and 4EBP1 in Y-Sesn2 KO mice (Expanded View Fig. 4D and E).

In aged WT mice that underwent sham surgery, AAV9-Sesn2 treatment had no effect on cardiac contractile functions (Fig. 5B) and expression levels of ANP and BNP in heart tissue (Fig. 5C). At 4 weeks after TAC, both EF and FS decreased; however, AAV9-Sesn2 transfection partially rescued the impaired cardiac contractile functions in aged WT mice (Fig. 5B). The increases in ANP and BNP mRNA levels were smaller in AAV9-Sesn2-treated aged WT mice than in AAV9-scram-treated aged WT mice; these mRNA expression levels remained higher than the levels observed in young WT mice (Fig. 5C). Immunoblotting analysis showed that AAV9-Sesn2 treatment rescued the levels of Sesn2 in aged WT mice that had been subjected to TAC or sham surgery (Fig. 5D and E). Moreover, we found that AAV9-Sesn2 treatment could attenuate the excessive activation of mTORC1, S6, and 4EBP1 in aged WT mice (Fig. 5D and E; Expanded View Fig. 5A and B). Thus, Sesn2 rescue can partially ameliorate pressure overload-induced cardiac hypertrophy induced in aged WT mice.

### 3.5 Rescue of age-related Sesn2 decline improves substrate metabolism and autophagy response

**Fig. 6:**
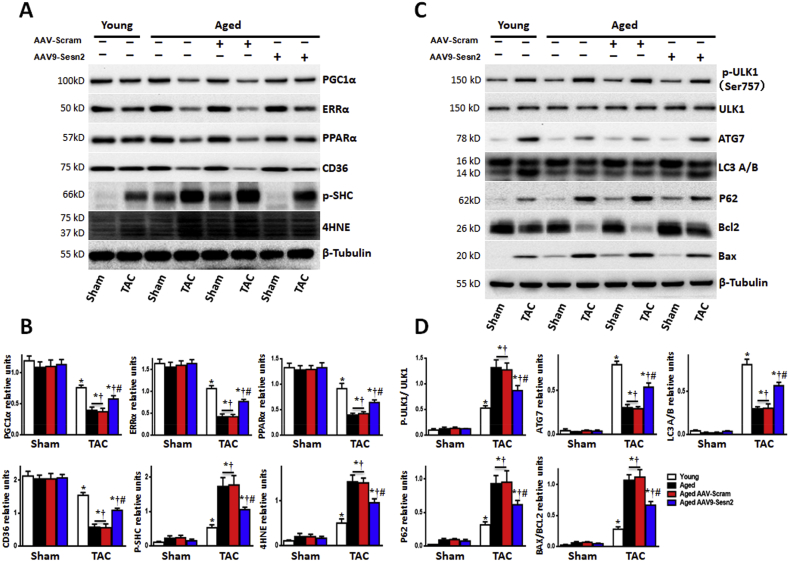
Sesn2 rescue in aged hearts improves myocardial metabolism and autophagy response to pressure overload. (A) Immunoblot for proteins in metabolic regulatory pathways from hearts of aged WT (Aged) mice and young WT (Young) mice at 4 weeks after TAC or sham surgery. (B) Quantification of relative expression levels of indicated proteins (n = 5–6 per group). (C) Immunoblot for indicated autophagy-related indicated proteins from hearts of aged WT and young WT mice at 4 weeks after TAC or sham surgery. (D) Quantification of relative expression levels of indicated proteins (n = 5–6 per group). Values are mean ± SEM, *p < 0.05 vs. Sham group; ^#^p < 0.05 vs. corresponding young WT group; ^†^p < 0.05 vs. corresponding Y-Sesn2 KO group.

Immunoblotting results suggested that the impaired expression levels of mitochondrial synthesis-associated proteins (PGC1α, ERRα, PPARα, and CD36) could be partially rescued by AAV9-Sesn2 treatment in aged WT (Aged) mice that had been subjected to pressure overload-induced cardiac hypertrophy. Sesn2 rescue led to reduced expression of oxidative stress-related proteins (i.e., SHC and 4HNE) in aged WT mice, but these levels remained higher than the levels observed in young WT (Young) mice (Fig. 6A and B). Overall, AAV9-Sesn2 treatment inhibited oxidative stress in aged WT mice that had been subjected to pressure overload-induced cardiac hypertrophy.

Sesn2 rescue in aged heart reduced the degree of pressure overload-induced phosphorylation of ULK1 at Ser^757^, but this phosphorylation occurred to a greater extent than that observed in young WT mice. The expression levels of autophagy-related proteins were partially rescued by AAV9-Sesn2 treatment in aged WT mice during pressure overload-induced cardiac hypertrophy, while expression levels of apoptosis-related proteins decreased after AAV9-Sesn2 treatment (Fig. 6C and D). These findings indicated that Sesn2 rescue could ameliorate the intolerance of aged hearts to pressure overload-induced hypertrophy.

## 4 Discussion

In this study, we systematically investigated the role of Sesn2 in myocardial hypertrophy; the results showed that Sesn2 inhibits myocardial hypertrophy by inhibiting the mTORC1 pathway, thereby regulating protein synthesis, metabolism, autophagy, and apoptosis. There were three important findings: 1) Pressure overload induced the expression of Sesn2, which inhibited cardiac hypertrophy by suppressing mTORC1 activity. 2) Compared with young WT mice, aged WT mice showed more severe cardiac insufficiency in pressure overload-induced cardiac hypertrophy; this was primarily because the expression of Sesn2 was reduced in aged heart, which led to weakened inhibition of mTORC1. 3) Sesn2 inhibits the activation of mTORC1 by interacting with components of the GATOR2 complex (MIOS, WDR24, and WDR59) during cardiac hypertrophy. To the best of our knowledge, this is the first report regarding the mechanism by which Sesn2 inhibits mTOR in the context of cardiac hypertrophy.

It has been controversial whether myocardial hypertrophy under stress is beneficial [45]. The main function of myocardial hypertrophy is to provide support for hypertrophic growth by increasing protein synthesis; however, persistent hypertrophy can cause heart failure or sudden death [46]. mTORC1 is essential for cell growth; in particular, appropriate mTORC1 activity and controlled induction of myocardial hypertrophy are important for a normal physiologic response to pathological stress within the heart. Pressure overload-induced stress can activate mTORC1, thereby causing myocardial hypertrophy to maintain normal wall tension; however, continuous pressure overload-induced stress may cause cardiac insufficiency. Thus far, myocardial hypertrophy is generally improved by drugs or transgenes that inhibit the activity of mTORC1 substrate [11]. Nonetheless, during pressure overload-induced cardiac hypertrophy, complete inhibition of mTORC1 can lead to a fatal dilatation-cardiac phenotype and accelerate the development of heart failure; this has led to sudden death in mice [23,47]. In the present study, pressure overload induced the expression of Sesn2; the development of cardiac hypertrophy induced further increase in Sesn2 expression (Fig. 1), which modulated mTORC1 and inhibited 4EBP1 and S6, thus delaying myocardial hypertrophy. Importantly, we reversed the pressure overload-induced cardiac hypertrophy of Y-Sesn2 KO mice (Expanded View Fig. 4) and aged WT mice (partial reversal; Fig. 5) by AAV9-Sesn2 transfection. However, we did not observe significant improvement of cardiac function after administration of AAV9-Sesn2 to young WT mice (Expanded View Table 3). Therefore, we plan to construct Sesn2-overexpressing mice to further investigate the role of Sesn2 in cardiac hypertrophy.

Metabolism is an important factor in the progression of cardiac insufficiency due to cardiac hypertrophy. To maintain continuous contraction and relaxation, the heart requires a large quantity of ATP; most (70%–90%) is generated by fat oxidation, while some (10%–30%) is generated by glucose oxidation and metabolism of other substrates [5,9]. Our results suggested that both fatty acid and glucose oxidation were reduced in mice with pressure overload-induced cardiac hypertrophy, which is consistent with the findings of previous reports [10,43,48]. The results of another study suggested that glucose oxidation was elevated in mice with cardiac hypertrophy [49]; glycolysis caused by pressure overload-induced hypertrophy reportedly exhibited a greater increase, compared with glucose oxidation in the heart [48]. These previous data are consistent with our results in terms of damage to glucose oxidation. Compared with young WT mice that were subjected to pressure overload-induced cardiac hypertrophy, greater reduction of fatty acid oxidation was observed in the heart of aged WT mice with cardiac hypertrophy. Compared with young WT mice, increased mitochondrial damage was observed in aged WT mice and Y-Sesn2 KO mice, while fewer mitochondria were observed (Fig. 4E); moreover, expression levels of PGC1α, ERRα, and PPARα—proteins that are related to mitochondrial synthesis—were reduced in a manner consistent with the observed mitochondrial morphology. The results of prior studies support our conclusions [10,[50], [51], [52]]. Although mTORC1 reportedly promotes mitochondrial biogenesis and oxidative metabolism [53], continuous stress and deregulation of mTORC1 activation lead to inhibitory protein synthesis. Further deterioration of cell metabolism promotes cell senescence, ultimately resulting in organ dysfunction [14,20]. Glucose transporter 4 translocates to the sarcolemma and transverse tubule membrane under normal and pathological stimulation [54]; activation of AKT may lead to increased levels of glucose transporter 4 in cardiomyocytes [55]. Notably, there were no significant differences in cardiomyocyte-specific glucose transporter 4, glycolysis, or glucose uptake among aged WT mice, Y-Sesn2 KO mice, and young WT mice that had been subjected to pressure overload-induced cardiac hypertrophy; however, glucose oxidation was significantly lower in aged WT mice and in Y-Sesn2 KO mice than in young WT mice, due to the reduction of mitochondrial function.

mTORC1 has been shown to inhibit autophagy by phosphorylating ULK1/2 at Ser^757^; phosphorylation of ULK1/2 at Ser^757^ can inhibit formation of the ULK1/Atg13/FIP200 complex, which promotes the formation of autophagy [56]. Moreover, mTORC1 also has been shown to inhibit the initiation of autophagy by interacting with Atg7 [57]. Compared with young WT mice, mTORC1 exhibited excessive activation in aged WT and Y-Sesn2 KO mice with cardiac hypertrophy; this activation was associated with elevated expression of p-ULK1 (S757) and reduced expression of Atg7. Autophagy was partially restored in aged WT mice after overexpression of Sesn2 by AAV9 injection. Autophagy has been reported to increase myocardial loss during the removal of abnormal proteins or damaged organelles, thereby resulting in compensated hypertrophy to heart failure [58]. However, this finding does not contradict the results of our study, because it was observed in a model of complete inhibition of mTORC1. Complete inhibition of mTORC1 is expected to lead to excessive autophagy, increased myocardial loss, and cardiac insufficiency [23,47]. Although excessive autophagy can lead to myocardial loss, abnormal proteins and damaged organelles must be removed by maintenance of appropriate autophagy under stress. The importance of Sesn2 in maintaining this balance was highlighted in the present study. In addition, we found that myocardial apoptosis was more severe in aged WT and Y-Sesn2 KO mice with pressure overload-induced cardiac hypertrophy, compared with young WT mice that had been subjected to cardiac hypertrophy. The partial reversal of apoptosis by overexpression of Sesn2 in aged WT mice was consistent with the findings of a previously published report [59].

**Fig. 7:**
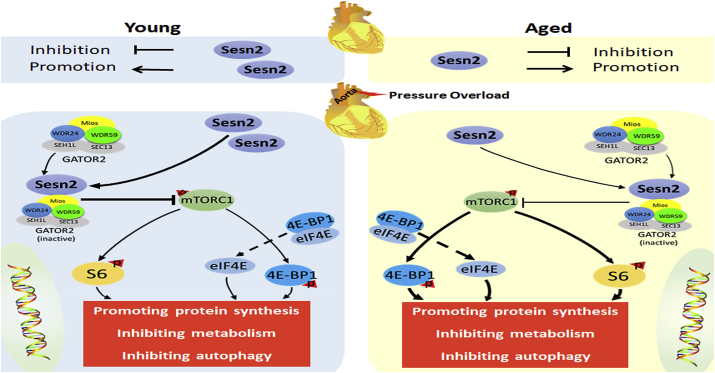
Molecular pathways involved in Sesn2 signaling. During pressure overload, Sesn2 expression increases. Elevated Sesn2 inactivates GATOR2 by binding interactions with its three subunits (MIOS, WDR24, and WDR59), thereby reducing the phosphorylation and activity of mTORC1. The mTORC1-induced phosphorylation of S6 and 4EBP1 decreases.

Sesn2 has been reported to inhibit mTORC1 through the AMPK–TSC2 pathway [27]. In our study, the phosphorylation levels of AMPK in young WT, aged WT, and Y-Sesn2 KO mice with cardiac hypertrophy increased after TAC, but there were no significant differences among the three groups; thus, Sesn2 may inhibit mTORC1 independent of the AMPK–TSC2 pathway. We found that Sesn2 interacted with MIOS, WDR24, and WDR59 of the GATOR2 complex to inhibit mTORC1 activity; this finding is consistent with the results of previous reports, in which Sesn2 inhibited mTORC1 [34,35]. Our proposed model for the molecular pathways involved in Sesn2 signaling, including stimuli that activate these pathways, is shown in Fig. 7. Although GATOR2 can inhibit mTORC1 through various mechanisms, the indirect inhibition of mTORC1 through binding interactions between Sesn2 and GATOR2 is consistent. In the context of cardiac hypertrophy, the mechanism by which GATOR2 inhibits mTORC1 is an important future research topic. In addition, there have been some reports regarding relationships among sestrin1, sestrin3, and mTOR [60,61]. However, when we measured the expression levels of sestrin1 and sestrin3, there were no significant differences among young WT, aged WT, and Y-Sesn2 KO mice (data not shown), suggesting that Sesn2 may be the key factor in the progression of cardiac hypertrophy.

This study had some limitations. First, the findings were not validated in human tissue samples or in cultures of human cells. In future studies, it would be useful to perform experiments in human pluripotent stem cells, using CRISPR-Cas9 genome engineering. Ideally, Sesn2 could be knocked out in human pluripotent stem cells, and a screening could then be performed for small molecules that rescue the functionality of cardiac myocytes. Furthermore, the findings could be supported by demonstrating changes in cardiac tissue from aged patients, compared with young patients; this could be achieved by performing qPCR analysis of cardiac tissue from a tissue bank. Second, we did not assess the percent survival of mice after treatment with AAV9-Sesn2. Therefore, it remains unclear whether restored expression of Sesn2 confers a survival benefit; other long-term (>8 weeks) phenotypic and molecular effects should also be investigated.

## 5 Conclusion

Our study showed that Sesn2 prevented pressure overload-induced cardiac hypertrophy by inhibiting mTORC1 through interactions with the GATOR2 complex. The reduction of Sesn2 expression was the main cause of cardiac hypertrophy in aged heart and may be a useful target for future drug and gene therapy treatment efforts.

## Declaration of competing interest

The authors do hereby declare that all illustrations and figures in the manuscript are entirely original and do not require reprint permission.

The authors declare that they have no conflicts of interest.
