# Supplementary material for: Substrate metabolism regulated by Sestrin2–mTORC1 alleviates pressure overload-induced cardiac hypertrophy in aged heart
Source: Redox Biol. 2020 Jul 9;36:101637. doi: 10.1016/j.redox.2020.101637 (PMC7363709; doi:10.1016/j.redox.2020.101637)
Supplement: Multimedia component 1 [file mmc1.docx]

**Substrate metabolism regulated by Sestrin2–mTORC1 alleviates pressure overload-induced cardiac hypertrophy in aged heart**

Nanhu Quan, Xuan Li, Jingwen Zhang, Ying Han, Weiju Sun, Di Ren, Qian Tong, Ji Li

**Expanded View Table 1:** Working heart function of WT(young and aged) and young Sesn2 KO mice subjected to sham surgery or TAC.

| **Parameter** | **Young WT** | | | **Aged WT** | | | **Young Sesn2 KO** | |
| --- | --- | --- | --- | --- | --- | --- | --- | --- |
|  | **Sham** | | **TAC** | **Sham** | | **TAC** | **Sham** | **TAC** |
| Systolic pressure (mmHg) | 88.26 ± 10.97 | 52.09 ± 6.53* | | 83.57 ± 8.80 | 47.21 ± 4.57* | | 84.76 ± 10.87 | 41.05 ± 8.35* |
| Diastolic pressure (mmHg) | 18.20 ± 1.42 | | 15.19 ± 1.85 | 18.90± 1.71 | 14.57 ± 1.34 | | 18.37 ± 1.51 | 13.78 ± 0.92 |
| CO (ml/min) | 10.60 ± 1.22 | | 6.39 ± 0.85* | 9.66 ± 0.89 | 4.69 ± 0.80* | | 9.78 ± 1.18 | 4.39 ± 0.77* |
| Coronary flow (ml/min) | 3.21± 0.28 | | 1.86 ± 0.25* | 2.86 ± 0.47 | 1.27 ± 0.13* | | 2.97 ± 0.53 | 1.19 ± 0.08* |
|  |  | | |  | | |  | |

Values are means ± SEM; **p*<0.05 *vs.* corresponding Sham group.

**Expanded View Table 2:** Echocardiographic assessment of cardiac function

| **Parameter (unit)** | **Young WT** | | **Young Sesn2 KO** | | **Aged WT** | | **Aged WT AAV9-sesn2** | |
| --- | --- | --- | --- | --- | --- | --- | --- | --- |
|  | **Sham** | **TAC** | **Sham** | **TAC** | **Sham** | **TAC** | **Sham** | **TAC** |
| Heart rate (min^-1^) | 423 ± 10 | 440 ± 12 | 420 ± 17 | 414 ± 12 | 429 ± 11 | 409 ± 14 | 429 ± 21 | 422 ± 39 |
| LVPWd (mm) | 0.80 ± 0.04 | 1.00 ± 0.03^#^ | 0.83 ± 0.05 | 1.16 ± 0.05^#.^* | 0.82 ± 0.04 | 1.15 ± 0.05^#.^* | 0.80 ± 0.07 | 1.09 ± 0.06^#^ |
| LVPWs (mm) | 1.01 ± 0.04 | 1.22 ± 0.04^#^ | 1.05 ± 0.04 | 1.36 ± 0.11^#^ | 1.01 ± 0.06 | 1.30 ± 0.06^#^ | 1.00 ± 0.08 | 1.27 ± 0.04^#^ |
| LVIDd (mm) | 3.56± 0.08 | 4.16 ± 0.10^#^ | 3.67 ± 0.06 | 5.03 ± 0.13^#.^* | 3.72 ± 0.06 | 4.97 ± 011^#.^* | 3.71 ± 0.09 | 4.53 ± 0.08^#^ |
| LVIDs (mm) | 2.66 ± 0.09 | 3.24 ± 0.13^#^ | 2.87 ± 0.10 | 3.87 ± 0.12^#.^* | 2.85 ± 0.09 | 3.72 ± 0.12^#.^* | 2.85 ± 0.11 | 3.51 ± 0.16^#^ |
| EF (%) | 67.15 ± 1.70 | 54.56 ± 1.65^#^ | 64.76 ± 1.65 | 36.93±1.65^#,^* | 64.58 ± 1.59 | 37.40± 1.51^#,^* | 60.98 ± 1.57 | 41.88± 2.06^#.^*† |
| FS (%) | 37.14 ± 1.34 | 28.14 ± 1.02^#^ | 35.29 ±1.28 | 17.86 ±0.94^#,^* | 35.21 ± 1.24 | 18.02 ±0.82^#,^* | 32.40 ± 0.28 | 22.83 ±0.57^#.^*† |

Values are means ± SEM; ^#^*p*<0.05 *vs.* corresponding Sham; **p*<0.05 *vs.* Young WT TAC group; †*p*<0.05 *vs.* Aged WT TAC group.

Abbreviations: TAC, transverse aortic constriction; AAV9, adeno-associated virus 9; LVPWd, left ventricular posterior wall at end-diastole; LVPWs, left ventricular posterior wall at end-systole; LVIDd, left ventricular internal diameter at end-diastole; LVIDs, left ventricular internal diameter at end-systole; EF, ejection fraction; FS, fractional shortening.

**Expanded View Table 3:** Echocardiographic assessment of cardiac function

| **Parameter (unit)** | **Young WT** | | **Young WT AAV9-sesn2** | |
| --- | --- | --- | --- | --- |
|  | **Sham** | **TAC** | **Sham** | **TAC** |
| Heart rate (min^-1^) | 423 ± 10 | 440 ± 12 | 435 ± 25 | 451 ± 33 |
| LVPWd (mm) | 0.80 ± 0.038 | 1.00 ± 0.03^#^ | 0.81 ± 0.07 | 1.01 ± 0.08^#.^* |
| LVPWs (mm) | 1.01 ± 0.04 | 1.22 ± 0.04^#^ | 0.98 ± 0.05 | 1.17 ± 0.10^#^ |
| LVIDd (mm) | 3.56± 0.08 | 4.16 ± 0.10^#^ | 3.55 ± 0.21 | 4.18 ± 0.15^#.^* |
| LVIDs (mm) | 2.66 ± 0.09 | 3.24 ± 0.125^#^ | 2.59 ± 0.19 | 3.29 ± 0.26^#.^* |
| EF (%) | 67.15 ± 1.70 | 54.56 ± 1.65^#^ | 67.21 ± 3.94 | 52.17±2.74^#,^* |
| FS (%) | 37.14 ± 1.34 | 28.14 ± 1.02^#^ | 38.69 ±1.97 | 30.19 ±1.29^#,^* |

Values are means ± SEM; ^#^*p*<0.05 *vs.* corresponding Sham group; **p*<0.05 *vs.* Young WT Sham group.

Abbreviations: TAC, transverse aortic constriction; AAV9, adeno-associated virus 9; LVPWd, left ventricular posterior wall at end-diastole; LVPWs, left ventricular posterior wall at end-systole; LVIDd, left ventricular internal diameter at end-diastole; LVIDs, left ventricular internal diameter at end-systole; EF, ejection fraction; FS, fractional shortening.


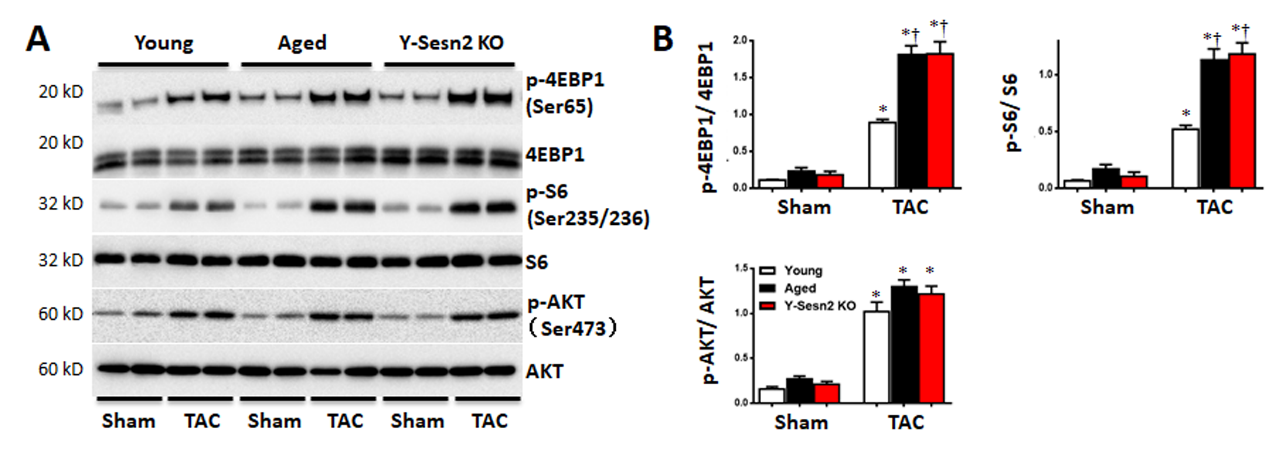


**Expanded View Figure 1. Aged WT and Y-Sesn2 KO hearts show similar responses to pressure overload induced by transverse aortic constriction (TAC).** (A) Immunoblot for indicated proteins from hearts of Y-Sesn2 KO, aged WT (Aged), and young WT (Young) mice at 4 weeks after TAC or sham surgery. (B) Immunoblotting analysis of p-S6/S6 and p-AKT/AKT expression levels in Y-Sesn2 KO, aged WT, and young WT mice at 4 weeks after TAC or sham surgery (n=5–6 per group). Values are mean ± SEM, ^*^p<0.05 vs. Sham group; ^†^p<0.05 vs. corresponding young WT group.


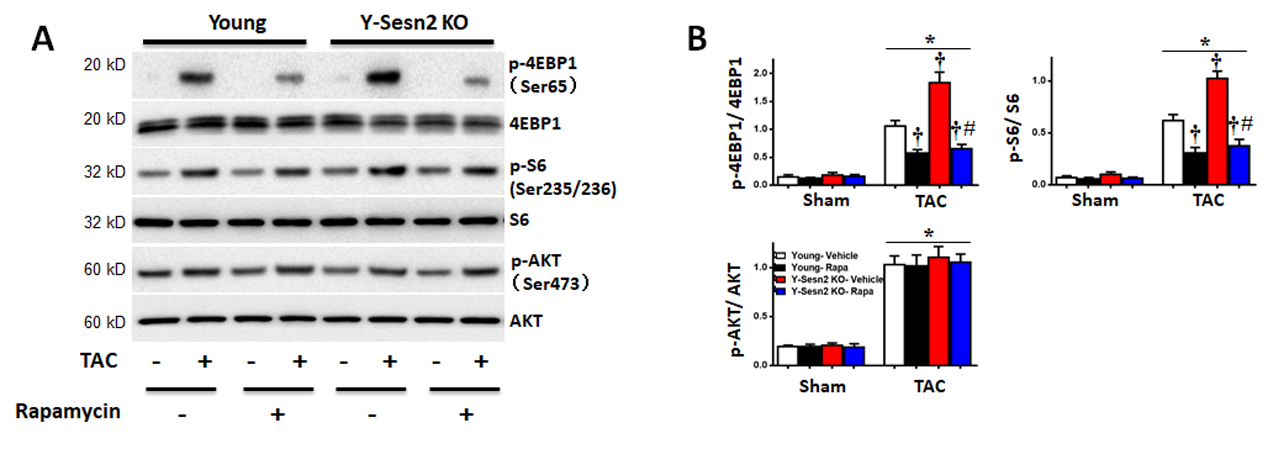


**Expanded View Figure 2. Inhibition of mTOR reduced cardiac hypertrophy induced by pressure overload.** (A) Immunoblot for indicated proteins from hearts of sham or transverse aortic constriction (TAC) operated young WT (Young) or Y-Sesn2 KO, treated with or without rapamycin. (B) Immunoblotting analysis of p-4EBP1/4EBP1, p-S6/S6, and p-AKT/AKT expression levels in sham or TAC operated young WT or Y-Sesn2 KO mice, treated with or without rapamycin (n=5–6 per group). Values are mean ± SEM, ^*^p<0.05 vs. Sham group; ^†^p<0.05 vs. corresponding young WT group; ^#^p<0.05 vs. corresponding Y-Sesn2 KO group.


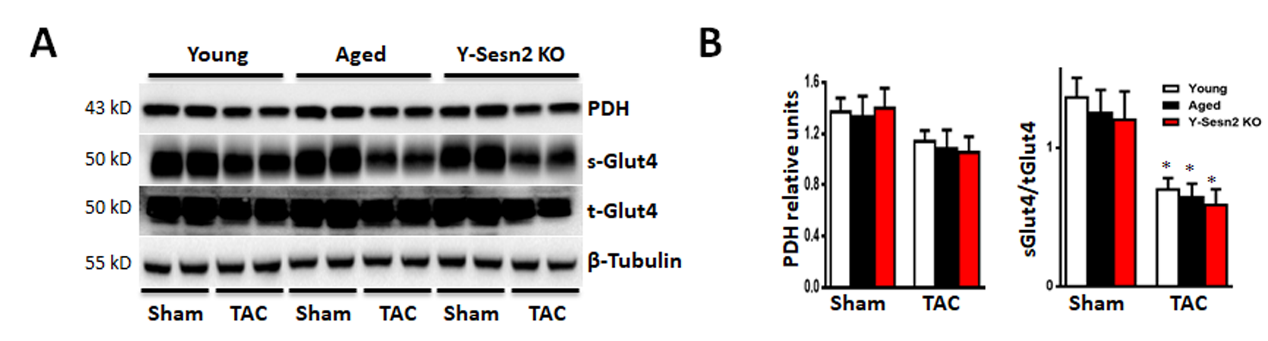


**Expanded View Figure 3.** **Regulation of substrate metabolism and autophagy in response to pressure overload.** (A) Immunoblot for pyruvate dehydrogenase (PDH) and glucose transporter 4 (Glut4) from hearts of Y-Sesn2 KO, aged WT (Aged), and young WT (Young) mice at 4 weeks after TAC or sham surgery. (B) Quantification of relative expression levels of PDH and Glut4 (n=5–6 per group). Values are mean ± SEM, ^*^p<0.05 vs. Sham group.


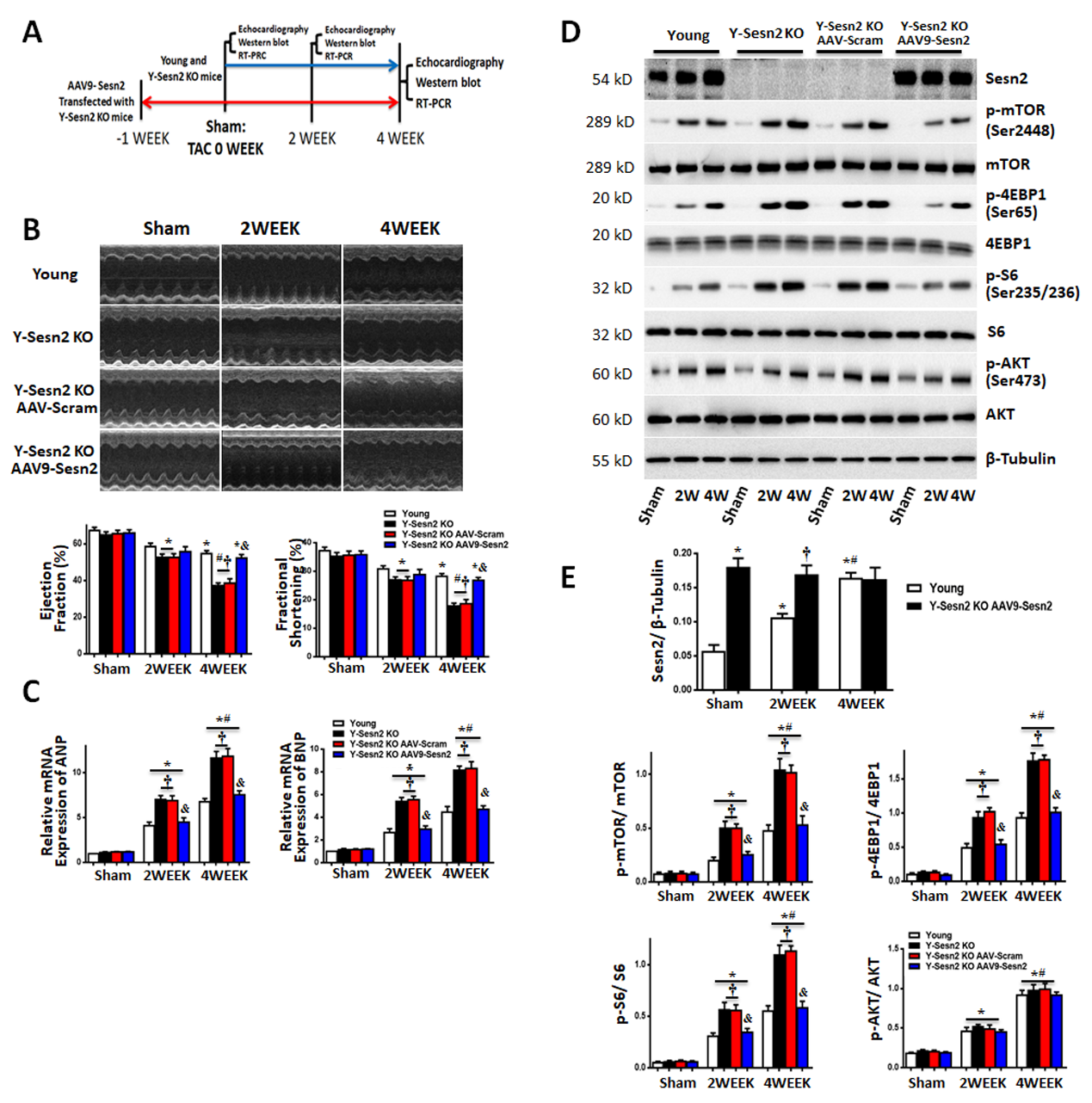


**Expanded View Figure 4. Rescue of impaired Sesn2 level supresses cardiac hypertrophy in Y-Sesn2 KO mice.** (A) Experimental design: Y-Sesn2 KO mice were treated with AAV9-Sesn2 at 1 week before TAC. Hearts of Y-Sesn2 KO and young WT (Young) mice were monitored by ultrasonography and heart tissues were harvested at indicated time points for immunoblotting and qPCR. (B) Echocardiography showed that recovery of impaired Sesn2 levels in Y-Sesn2 KO hearts improved resistance of Y-Sesn2 KO hearts to TAC, as shown by EF and FS (n=6–10 per group). (C) Relative expression analysis of ANP and BNP (n=4–5 per group). (D) Immunoblot for indicated proteins from hearts of sham or TAC operated young WT or Y-Sesn2 KO mice, treated with or without AAV9-Sesn2 as indicated in panel A. (E) Quantification of immunoblot shown in panel D (n=4–5 per group). Values are mean ± SEM, ^*^p<0.05 vs. Sham group; ^†^p<0.05 vs. corresponding young WT TAC group; ^#^p<0.05 vs. TAC 2 week group; ^&^p<0.05 vs. corresponding Y-Sesn2 KO group.


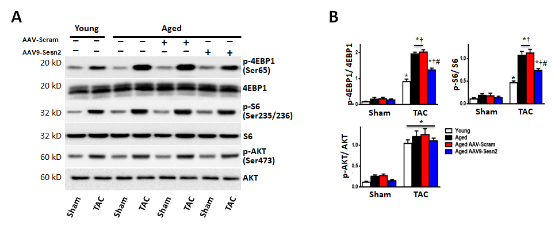


**Expanded View Figure 5. Sesn2 rescue in aged hearts ameliorates pressure overload-induced cardiac hypertrophy.** (A) Immunoblot for indicated proteins from hearts of sham or TAC operated young WT(Young) mice and aged WT (Aged) mice, treated with or without AAV9-Sesn2 as indicated in **Fig 5A**. (B) Quantification of immunoblot shown in panel A (n=5–6 per group). Values are mean ± SEM, ^*^p<0.05 vs. Sham group; ^#^p<0.05 vs. corresponding young WT group; ^†^p<0.05 vs. corresponding aged WT group.

**Expanded View Table 4. Antibodies and polymerase chain reaction primers used in this study.**

| **USE** | **Antibody** | **Supplier** | **Catalog No.** | **Dilution** |
| --- | --- | --- | --- | --- |
| **WB** | p-AMPK(Thr172) | Cell Signaling | #2535 | 1:1000 |
| **WB** | AMPK | Cell Signaling | #5831 | 1:1000 |
| **WB** | p-AKT(Ser473) | Cell Signaling | #4060 | 1:1000 |
| **WB** | AKT | Cell Signaling | #9272 | 1:1000 |
| **WB** | p-4EPB1(Ser65) | Cell Signaling | #9451 | 1:1000 |
| **WB** | 4EBP1 | Cell Signaling | #9644 | 1:1000 |
| **WB** | p-S6(Ser235/236) | Cell Signaling | #4858 | 1:2000 |
| **WB** | S6 | Cell Signaling | #2217 | 1:1000 |
| **WB** | p-mTOR(Ser2448) | Cell Signaling | #5536 | 1:1000 |
| **WB** | mTOR | Cell Signaling | #2983 | 1:1000 |
| **WB** | p-ULK1(Ser757) | Cell Signaling | #6888 | 1:1000 |
| **WB** | ULK1 | Cell Signaling | #8054 | 1:1000 |
| **WB** | Sestrin2 | Protein Tech | 10795-1-AP | 1:1000 |
| **WB** | PGC1α | Santa Cruz | SC13067 | 1:1000 |
| **WB** | ERRα | Novus Bio | NBP1-47254 | 1:2000 |
| **WB** | PPARα | ABCAM | ab32064 | 1:1000 |
| **WB** | CD36 | Novus Bio | NB400-144 | 1:1000 |
| **WB** | Atg7 | Cell Signaling | #2631 | 1:1000 |
| **WB** | LC3 A/B | Cell Signaling | #12741 | 1:1000 |
| **WB** | P62 | Cell Signaling | #5114 | 1:1000 |
| **WB** | Bcl2 | Cell Signaling | #3498 | 1:1000 |
| **WB** | Bax | Cell Signaling | #14796 | 1:1000 |
| **WB** | Mios | Cell Signaling | #13557 | 1:1000 |
| **WB** | WDR24 | Novus Bio | NBP2-20892 | 1:1000 |
| **WB** | WDR59 | Novus Bio | NBP1-82305 | 1:1000 |
| **WB** | 4HNE | Abcam | Ab46545 | 1:1000 |
| **WB** | p-SHC (Ser66) | Calbiochem | 566807 | 1:1000 |
| **WB** | β-Tubulin | Cell Signaling | #2146 | 1:1000 |
| **Primers for quantitative polymerase chain reaction (qPCR)** | | | | |
| **β-actin** | Forward | AGAGGGAAATCGTGCGTGAC | | |
|  | Reverse | CAATAGTGATGACCTGGCCGT | | |
| **PGC1α** | Forward | GTAAATCTGCGGGATGATGG | | |
|  | Reverse | AGCAGGGTCAAAATCGTCTG | | |
| **CPT1β** | Forward | GAGTTCTCGATGGCTTTCCG | | |
|  | Reverse | GACAGGACACTGTGTGGGTGAG | | |
| **MCAD** | Forward | CTAACCCAGATCCTAAAGTACCCG | | |
|  | Reverse | GGTGTCGGCTTCCAAATGA | | |
| **VLCAD** | Forward | ATGCAGTCGGCTCGGATGACCC | | |
|  | Reverse | TGAGAAATTGTGCCTGTTCTTC | | |
| **Primers for mitochondrial DNA (mtDNA) PCR analysis** | | | | |
| **mtDNA** | Forward | TGCTAGCCGCAGGCATTAC | | |
|  | Reverse | GGGTGCCCAAAGAATCAGAAC | | |
| **Ndufv1** | Forward | CTTCCCCACTGGCCTCAAG | | |
|  | Reverse | CCAAAACCCAGTGATCCAGC | | |
